# Effects of a Fundamental Motor Skill-Based Afterschool Program on Children’s Physical and Cognitive Health Outcomes

**DOI:** 10.3390/ijerph17030733

**Published:** 2020-01-23

**Authors:** Joonyoung Lee, Tao Zhang, Tsz Lun (Alan) Chu, Xiangli Gu, Ping Zhu

**Affiliations:** 1 Department of Kinesiology, Health Promotion and Recreation, University of North Texas, Denton, TX 76203, USA; joonyounglee@my.unt.edu; 2 Department of Psychology, University of Wisconsin-Green Bay, Green Bay, WI 54311, USA; chua@uwgb.edu; 3 Department of Kinesiology, The University of Texas at Arlington, Arlington, TX 76019, USA; Xiangli.Gu@uta.edu; 4 Department of Educational Psychology, University of North Texas, Denton, TX 76203, USA; PingZhu@my.unt.edu

**Keywords:** motor skill performance, moderate to vigorous physical activity, cognitive functioning, school-aged children, afterschool program

## Abstract

Globally, more than half of school-aged children do not engage in the recommended 60 minutes of daily moderate to vigorous physical activity (MVPA). Given that developing sufficient fundamental motor skills (FMS) competence during early elementary school years is important for a child’s physical and cognitive development, the purpose of this study was to examine the effects of an 8-week FMS-based afterschool program on physical and cognitive health outcomes among elementary children. Participants were 31 K–2 students (19 girls, 12 boys; *M*age = 6.65 ± 0.98) from three public elementary schools in the southwestern United States who were assigned to the intervention group (FMS-based afterschool program; *n* = 20) or the control group (traditional afterschool program; *n* = 11). A 2 × 2 repeated measures MANOVA showed significant changes in FMS competence and MVPA between the intervention and the control group over time. However, no significant changes were found in cognitive functioning. The 8-week FMS-based afterschool program showed significant improvements in FMS competence and MVPA, compared to a traditional afterschool program. This finding suggests that structured FMS-focused strategies (e.g., fun games and goal setting) can be a critical component when implementing a physical activity program to enhance children’s motor skills and physical activity behavior.

## 1. Introduction

Globally, more than half of school-aged children do not engage in the recommended 60 minutes of daily moderate to vigorous physical activity (MVPA) [1] and the childhood obesity rate has increased from 13.9% in 2000 to 18.4% in 2016 in the United States [2]. Research indicates that physical inactivity and sedentary behaviors are significant correlates of childhood obesity [3,4]. In addition, childhood obesity and physical inactivity could result in serious adverse health consequences such as cardiovascular disease [5], type 2 diabetes [6], asthma [7], sleep apnea [8], depression [9], and psychosocial issues [10].

A growing body of research has revealed that fundamental motor skills (FMS) competence during childhood are important correlates of obesity, and contribute to children’s physical activity participation and cognitive health [11,12,13]. Specifically, FMS competence has been considered as the building blocks to develop and perform complex movement skills required for sufficient participation in physical activity across the lifespan [13,14] including locomotor skills (e.g., running, galloping, and jumping) and object control skills (e.g., dribbling, catching, and throwing). Stodden and colleagues [13] proposed a conceptual model indicating that developing sufficient FMS competence in childhood may increase the possibilities for children to engage in regular physical activity and influence the trajectory of childhood obesity. Cross-sectional studies support that children’s FMS competence is associated with higher levels of physical activity [15], less sedentary behavior [16], higher cardiorespiratory fitness [14], and better weight status [17]. A recent systematic review also indicated that developing sufficient FMS allows children to function effectively and independently for their physical, social, and cognitive growth [18]. Early elementary school years are a crucial time for developing FMS competence as they establish physical activity habits in children’s future development, and childhood is an ideal age period to benefit from quality training and education with regard to motor skill learning [14,19]. 

Structured FMS-focused programs (instruction/lesson) and unstructured activities (child free-play) may be an effective avenue for encouraging children to engage in various movement skills in early childhood years (i.e., age 3–8 years [20]). However, unstructured activities can only provide children with opportunities to engage in physical activities, and do not encourage learning FMS [21]. Previous FMS intervention studies have shown significant improvement of FMS competence (i.e., locomotor and object control skills) among school-aged children, ranging from 3 to 10 years old [20,22,23,24]. For instance, Bakhtiari and colleagues [22] applied an eight-week period of selected exercises (24 lessons; three days per week; each session lasted 45 minutes) focusing on FMS competence for 9-year-old girls. The findings demonstrated that girls in the intervention group significantly improved locomotor, object control, and total motor skills compared to the control group. However, the intervention study only focused on the effects of an FMS program on children’s motor competence, and specific information about the FMS program was not provided (e.g., type of activities, time to intervene; [22]). Therefore, it is imperative to provide a more elaborate procedure and information for the FMS program. Further study is needed to accurately describe the FMS program so that schools, researchers, and/or practitioners can implement the FMS program to enhance children’s physical activity participation and motor skill development, which may also contribute to children’s cognitive improvement [25].

Positive relationships between physical activity and cognitive performance have been established in recent systematic reviews [25,26]. For example, Donnelly and colleagues [25] found that MVPA participation enhanced the brain’s natural capacity for plasticity, contributing to enhanced cognitive functioning. However, most studies regarding the benefits of physical activity on cognitive functioning have focused primarily on older adults. These studies have demonstrated that aerobic exercise training positively affected the brain function and cognitive performance of older adults [27]. To date, limited intervention studies have examined the effects of FMS competence on physical activity and cognitive functioning among children, and the underlying behavioral mechanism is not clear [25]. Gu and colleagues [28] provided preliminary cross-sectional evidence that FMS competence, especially object control skills, are significant predictors of MVPA and cognitive functioning among minority children. The results suggest that structured skill-based physical activity programs should be emphasized in childhood education, which may promote children’s overall well-being.

Unfortunately, children’s physical inactivity and sedentary behaviors during school time have increased from the school curriculum in recent years [29]. In addition, children’s fundamental movement patterns may develop over time, but mastery of FMS would not increase naturally and requires instruction and practice [19,30]. Therefore, structured afterschool programs throughout the elementary years may provide children with opportunities to practice FMS and enhance physical activity behavior and cognitive development. Guided by Stodden and colleagues’ [13] conceptual model, the present study aimed to examine the effects of an eight-week FMS-based afterschool program on physical and cognitive health among K–2 elementary school children. It was hypothesized that children who participated in a FMS-based afterschool program would demonstrate better FMS competence, higher MVPA during school, and better cognitive functioning across time than their counterparts in the control condition (the regular afterschool program).

## 2. Materials and Methods

### 2.1. Participants

Thirty-eight K–2 students from three public elementary schools in the same school district in the southwestern USA participated in this study. However, seven students’ data were excluded in the final data analysis due to missing and incomplete assessments (i.e., FMS competence, physical activity, cognitive functioning). Thus, the final participants were 31 children (*M*age = 6.65 ± 0.98; 61% girls) in this study. The university’s institutional review board reviewed and approved the study protocol before the data collection (Project identification code: 16-357). Informed parental consent and children’s assent forms were obtained in accordance with the institutional review board, school district requirements, and the Declaration of Helsinki prior to data collection.

### 2.2. Intervention Procedure

Based on the school district’s recommendation and convenience, the researchers assigned participants at the school level to one of two groups: the intervention (1 school, *n* = 20) or the control group (2 schools, *n* = 11). During the eight-week intervention according to a previous study [22], children in the intervention group (13 girls, 7 boys) participated in the FMS intervention embedded in the afterschool program (3:30 pm–4:30 pm) three times per week (60 minutes each time) in 24 sessions (Appendix A, Table A1), while children in the control group (6 girls, 5 boys) followed a regular afterschool program (e.g., unstructured child free-play, drawing, reading, and/or academic tutoring). The typical afterschool programs (3:00 pm–6:00 pm) in the schools were provided by the school district without additional motor skill-related instructions. Typically, due to their job situations and duties, the children’s parents cannot pick up their children right after school, so they pay and sign their children up for the afterschool program, and pick them up at the end of the afterschool program, sometime between 5:00 pm and 6:00 pm.

The FMS intervention aimed to promote FMS competence by focusing on the mastery of 12 basic motor skills: running, hopping, galloping, leaping, jumping, sliding, striking, kicking, dribbling, catching, overhand throwing, and underhand rolling. Each intervention session lasted 60 minutes and included three activity parts: (a) 10 minutes of instruction and preparation, (b) 45 minutes of skill instruction and practice, and (c) 5 minutes in a closing activity. During the 45-minute motor skill practice, participants were divided into two groups based on the low and high motor skills competence assessed in this study, and were encouraged to practice with various tasks (e.g., cooperative and self-competition games, independent and goal-driven activities). In addition, as one of the instructional strategies, we reinforced the students’ goal setting including encouraging students to reach personal goals (e.g., “How many times can you hit the target of the wall by overhand throwing a ball in five minutes?” [31]). To ensure that children engaged in the same exercise duration and intensity, stopwatches and field observations were used to check the fidelity of the intervention. Two well-trained graduate research assistants, who had more than two years of teaching experience, served as physical activity specialists; they led the FMS-based afterschool program and recorded the participants’ attendance in the intervention log. Additionally, the graduate research assistants were trained and obtained child cardiopulmonary resuscitation (CPR) and First Aid certification for any emergencies during the intervention.

### 2.3. Instrumentation 

#### 2.3.1. Anthropometric Assessment 

A Health-o-meter 500KL digital physician height/weight scale (Pelstar, LLC, St. McCook, IL) was used to measure the children’s height and weight (without shoes) to compute BMI using the following formula: BMI = (weight [kg]/height^2^ [m^2^]). The anthropometric assessments were taken at each site for each child by trained research assistants.

#### 2.3.2. Fundamental Motor Skills (FMS) Competence

Children’s FMS competence was assessed using the Test of Gross Motor Development, 2^nd^ edition(TGMD-2, Ulrich, 2000; [32]) before and after the 8-week intervention. The TGMD-2 includes two skill categories: six locomotor skills (i.e., galloping, hopping, leaping, running, horizontal jumping, and sliding) and six object control skills (i.e., dribbling, catching, kicking, striking, overhand throwing, and underhand rolling). For the test evaluation, each skill was categorized into 3–5 components, and each component was scored as either 1 (present) or 0 (absent). The children’s FMS competence test was conducted in each school’s indoor gymnasium after obtaining the school administrators’ permission. Two motor skill subset scores (locomotor and object control skills) were computed from the sum of raw scores from each subset. Two trained examiners rated the children’s FMS competence and achieved 91% interrater reliability (0.89 for locomotor skills and 0.92 for object control skills, respectively). The TGMD-2 is a valid and reliable assessment tool for measuring school-aged children’s (aged 3–11) FMS competence and shows good test–retest reliability and internal consistency (ICC = 0.92 for locomotor skills; ICC = 0.97 for object control skills; [32]).

#### 2.3.3. Moderate to Vigorous Physical Activity

Children’s MVPA data were collected with Actical accelerometers (Mini-Mitter Co., Inc., Bend, OR) using an epoch length of 60 seconds during school hours (8:00 am–3:00 pm) for five consecutive days in the same week. We accept that this large epoch length may underestimate MVPA [12,29]. In addition, children’s MVPA was measured only during the school time due to practical limitations (i.e., losing or missing devices; participant compliance). The accelerometer is an objective tool that provides researchers and practitioners with information regarding the frequency, intensity, and duration of physical activities [33]. With the guidance of trained graduate research assistants, the participants wore accelerometers with an elastic band on the non-dominant hand in the morning and took the band off in the afternoon. Only children with ≥4 hours of valid wear time during school hours on ≥3 days were included in the analyses [12]. The Actical activity monitor was shown to have good reliability and validity for measuring physical activity in children [34].

#### 2.3.4. Cognitive Functioning

Cognitive functioning among the children was measured using the parent proxy-report format of the Pediatric Quality of Life Inventory^TM^ (PedsQL^TM^ 4.0, Varni et al., 2011) Cognitive Functioning Scale for children (5–7 years; [35]). The parent report format measures a parent’s perception of the child’s cognitive functioning by asking how much of a problem each item has been in the past month. The questionnaire comprises six items (e.g., “In the past one month, how much of a problem with difficulty remembering what people tell him/her has this been for your child?”) with a 5-point Likert scale, ranging from 0 (never a problem) to 4 (almost always a problem); it demonstrated sufficient reliability and validity in previous studies [35]. The six item scores were reversed and scaled to 100 (0 = 100, 1 = 75, 2 = 50, 3 = 25, 4 = 0), with higher scores indicating fewer occurrences of cognitive problems. The average of all six recoded scores was computed as the mean cognitive functioning score. The internal reliability for the six-item scale was high (α = 0.89) in the present study.

### 2.4. Data Analysis

After screening the raw data for missing data, normality, and outliers, the data analysis was conducted using SPSS 25.0 for Windows (IBM Corp., Armonk, NY). The pre- and posttest data related to FMS competence and physical and cognitive health outcomes (i.e., MVPA and cognitive functioning, respectively) were analyzed. In this study, independent-sample t-tests were used to examine any group differences prior to the intervention. A 2 × 2 repeated measures multivariate analysis of variance (MANOVA) involving all dependent variables (i.e., FMS competence, MVPA, and cognitive functioning) was used to examine the intervention effect, with group (intervention vs. control) as the between-subjects variable, and time (baseline vs. post-intervention) as the within-subjects variable [36]. In other words, the 2 × 2 repeated MANOVA was employed to investigate the effects of the 8-week FMS-based afterschool program on the FMS competence, MVPA during school hours, and cognitive functioning. Follow-up univariate and Bonferroni post hoc tests were used to examine group differences. Partial η values of 0.01, 0.09, and 0.25 were used to indicate the small, medium, and large effect sizes, respectively, in the multivariate analyses. The criteria for Cohen’s *d* were 0.20 (small), 0.50 (medium), and 0.80 (large), representing the effect sizes for group differences [37].

## 3. Results

### 3.1. Baseline Descriptive Characteristics between Groups 

The descriptive characteristics of the participants from the baseline are shown in Table 1. The independent-sample t-test demonstrated no statistically significant differences between the groups from the baseline on age [*t* (29) = −0.72, *p* = 0.47], height [*t* (29) = 0.01, *p* = 0.99], weight [*t* (29) = −1.15, *p* = 0.26], BMI [*t* (29) = −1.87, *p* = 0.07], locomotor skills [*t* (29) = −1.44, *p* = 0.16], object control skills [*t* (29) = −0.63, *p* = 0.53], total motor skills [*t* (29) = −1.09, *p* = 0.28], MVPA [*t* (29) = −1.24, *p* = 0.30], and cognitive functioning [*t* (29) = −0.37, *p* = 0.71]. 

### 3.2. Multivariate Analysis of Variance

The MANOVA results indicated that there was significant changes in physical and cognitive health outcomes between the intervention and the control groups over time [*F* (4, 26) = 16.839, *p* < 0.001, partial η2 = 0.721]. Univariate tests further indicated significant changes in locomotor skills [*F* (1, 29) = 23.430, *p* < 0.001, partial η2 = 0.447], object control skills [*F* (1, 29) = 40.517, *p* < 0.001, partial η2 = 0.583], total motor skills [*F* (1, 29) = 46.277, *p* < 0.001, partial η2 = 0.615], and MVPA [*F* (1, 29) = 15.326, *p* = 0.001, partial η2 = 0.346], but not in cognitive functioning [*F* (1, 29) = 0.141, *p* = 0.710, partial η2 = 0.005]. Table 2 shows the means and standard deviations for the baseline and post-intervention scores on the motor skills, MVPA, and cognition functioning. 

### 3.3. Bonferroni Post Hoc Tests 

**Figure 1:**
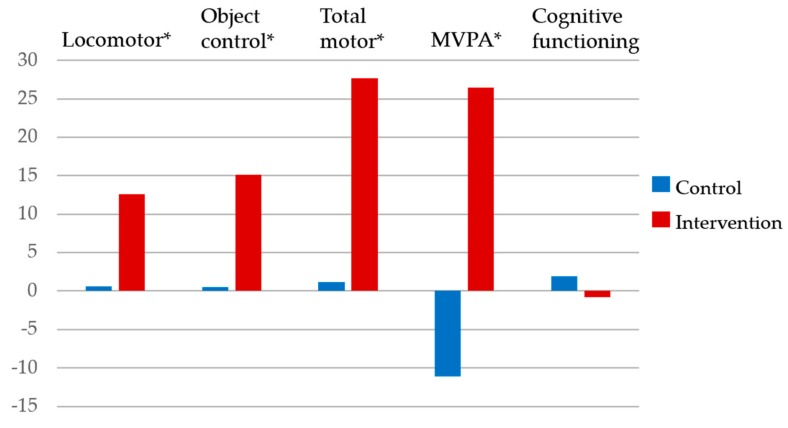
Gain scores in locomotor skills, object control skills, total motor skills, MVPA, and cognitive functioning. * indicates significant interaction effects (*p* ≤ 0.05).

Bonferroni post hoc tests indicated the significant group × time effects (*ps* < 0.05) on locomotor skills (intervention: *M*T1 = 25.4 vs. *M*T2 = 37.98, *d* = 1.88; control: *M*T1 = 29.73 vs. *M*T2 = 30.32, *d* = 0.05), object control skills (intervention: *M*T1 = 24.68 vs. *M*T2 = 39.78, *d* = 1.68; control: *M*T1 = 27.05 vs. *M*T2 = 27.59, *d* = 0.05), total motor skills (intervention: *M*T1 = 50.08 vs. *M*T2 = 77.75, *d* = 1.93; control: *M*T1 = 56.77 vs. *M*T2 = 57.91, *d* = 0.05), and MVPA (intervention: *M*T1 = 143.62 vs. *M*T2 = 170.06, *d* = 0.65; control: *M*T1 = 166.24 vs. *M*T2 = 155.17, *d* = 0.19), but not on cognitive functioning (*p* > 0.05). Figure 1 indicates the gain scores for each outcome in the present study.

## 4. Discussion

The major purpose of this study was to investigate the eight-week FMS-based afterschool program on children’s physical and cognitive health outcomes including FMS competence, MVPA, and cognitive functioning among elementary school children. After the eight-week FMS intervention, the intervention group showed significant improvements in FMS competence and MVPA relative to the control group, but no significant improvements were observed in cognitive functioning. The control group did not display any enhancements over time in the present study. 

The changes of FMS competence are broadly consistent with findings from previous research [22,24,38], which suggests that a structured FMS-emphasized activity program may benefit children’s FMS competence in both locomotor and object control skills. However, some physical education (PE)-based intervention studies found that only locomotor skills, and not object control skills, increased after the intervention among children. For instance, de Araujo and colleagues [23] indicated that PE settings including several weekly vigorous sports (i.e., skateboarding, roller skating, climbing, and parkour activities), significantly improved locomotor skills when compared to traditional PE classes, but no changes in object control skills were noted. In addition, McKenzie and colleagues [39] found no group differences in object control skills between an intervention program embedded in PE classes and a control condition, even though both groups increased locomotor skills competence. Furthermore, Boyle-Homes and colleagues [40] conducted a study using a developmental PE curriculum—Michigan’s Exemplary Physical Education Curriculum (EPEC)—with PE teachers’ assessment and feedback and showed improvements in school-aged children’s motor skills. The efficacy of developing FMS competence may depend on the structured FMS strategies because FMS itself cannot be expected to promote an effective learning environment [41]. PE-based interventions may contribute to overall FMS competence among children if the curriculum highlights FMS development instead of general activities or free play [42].

The afterschool program may not only provide children with practice with FMS, but also influence physical activity behavior in a school [4]. This FMS intervention during the afterschool program helped contribute to daily MVPA, although our participants were already highly active from the baseline (>2 hours of MVPA during the school time) compared to the general population of sedentary children (<1 hour of MVPA daily [1]). Gortmaker and colleagues [43] demonstrated the longitudinal effects of an afterschool physical activity intervention program on children’s physical activity levels with an environmental change approach (i.e., environmental change, educational activities, and parent engagement). Although the current study did not apply an environmental change approach while emphasizing learning and practicing FMS in the afterschool program, significant changes of FMS competence and physical activity level during the school hours were observed over time in the intervention group. This line of research also has implications for current perceived barriers in school PE (e.g., intensely focusing on academic achievement, lack of school support, lack of financial support, and poor quality teaching [44]).

The findings of the present study did not support the relation between FMS competence and cognitive functioning, showing no significant improvement in cognitive functioning after the eight-week afterschool program in the current study compared to the previous cross-sectional study. Gu and colleagues [28] examined the association between FMS competence and cognitive functioning among Hispanic kindergarteners (*M*age = 5.37) with a cross-sectional design, the results of this study might yield inconsistent findings with their study. Consistent with the findings of a previous intervention study (motor skill-based program), Krafft et al. [45] examined the impact of community-based motor skill intervention on elementary school children’s cognitive functioning as measured by the Cognitive Assessment System (CAS). They applied an afterschool exercise program (instructor-led aerobic activities; tag and jump rope) for 40 minutes per day for eight months in a public health community center, but the results showed no significant changes over time in cognitive functioning among the children in the intervention group. On the other hand, van der Niet and colleagues [46] noted that a recess-based 22-week physical activity program (30 minutes during lunch recess, twice a week; required cognitive effort in physical activity games) significantly improved the cognitive performance of elementary school children (8–12 years old) when compared with a control group. The inconsistent results might be due to the different tool used to examine cognitive functioning or different age groups. For instance, van der Niet and colleagues’ [46] study measured students’ cognitive (executive) functioning using self-assigned tests (i.e., Stroop test, measuring inhibition, digit span test) compared to parent reports. Further research is needed to identify an evidence-based FMS program to promote children’s cognitive functioning. Research [47,48] has also suggested that integrating cognitive engagement into physical activity is necessary to establish the foundation for children to improve their cognitive processes and functioning. Therefore, FMS interventions including cognitively engaging activities such as exergaming should be included in future intervention studies. Overall, this line of research is still in its infancy; thus, more investigation is needed to examine the influence of the FMS intervention on cognitive functioning among children. 

Despite the merits and contributions of this study, several limitations remain. First, although our small sample size was comparable to samples used in other elementary motor skill intervention studies [22,23], the results of this study might not be generalized beyond the population of the current study. Second, a different sample size of girls and boys in the intervention group may affect the findings and limit the investigation due to differences in FMS competence based on sex [49]; therefore, future intervention research will be required to recruit balanced participants for boys and girls. Third, since the participants in our study were already highly active children compared to the general population [1], we cannot generalize these findings to sedentary children. Fourth, using the parent proxy-report to assess children’s cognitive functioning may provide a subjective perception and assessment. Future studies should use a more objective way to measure cognitive functioning (e.g., Stroop test, digit span test, visual memory span, trail making test). Fifth, although objective measures of school MVPA time was the strength of this study, we did not measure physical activity outside of the school setting (e.g., leisure times in community and home settings) due to the practical limitations (i.e., losing or missing devices; participant compliance). Therefore, our findings may not be representative of a child’s usual physical activity behavior. It would be of interest to identify the effects of FMS interventions on physical activities for both within-school and out-of-school settings in the future. Several studies have suggested that physical activity intervention programs before school [50] and during recess time [51] are beneficial for increasing children’s physical activity. Future research is warranted to investigate the effect of FMS intervention embedded in afterschool programs compared to other school programs such as before-school and recess interventions. Finally, to examine the long-term effect of the FMS intervention, implementing a longitudinal intervention (e.g., nine months) with three- or six-month follow-up measures is recommended for future studies.

## 5. Conclusions 

The findings of this study showed that a structured FMS-based afterschool program was more efficacious in improving both FMS competence and physical activity among children than the traditional afterschool programs. These findings provide empirical evidence, demonstrating the effectiveness of a structured motor skill program in the afterschool setting, which has positive influences on children’s FMS competence and physical activity behavior during school time. School administrators and teachers should consider developing and designing structured FMS-based programs including developmentally appropriate fun games (activities) and goal-setting strategies to promote children’s learning outcomes (i.e., psychomotor, cognitive, and affective). Further research is needed to apply FMS-based programs in other school settings such as during recess and classroom breaks, which can contribute to children’s FMS learning experiences and physical activity behaviors by PE and classroom teachers as facilitators. Most importantly, school practitioners need to be aware of the importance of developing FMS competence in children because FMS provide the foundation for physical development.

## Figures and Tables

**Table 1:** Descriptive characteristics between the groups from the baseline.

Variable	Control (*n* = 11)	Intervention (*n* = 20)	*t*	*p*
Age, *M* ± *SD*	6.82 ± 1.25	6.55 ± 0.82	−0.72	0.47
Sex (female/male), *n*	6/5	13/7	N/A	N/A
Anthropometry				
Height (cm), *M* ± *SD*	121.58 ± 7.96	121.61 ± 7.77	0.01	0.99
Weight (kg), *M* ± *SD*	26.14 ± 4.53	24.11 ± 4.78	−1.15	0.26
BMI (kg/m^2^), *M* ± *SD*	17.63 ± 2.2	16.21 ± 1.92	−1.87	0.07
FMS competence				
Locomotor skills, *M* ± *SD*	29.73 ± 7.96	25.40 ± 8.02	−1.44	0.16
Object control skills, *M* ± *SD*	27.05 ± 10.79	24.68 ± 9.73	−0.63	0.53
Total motor skills, *M* ± *SD*	56.77 ± 16.72	50.08 ± 16.11	−1.09	0.28
MVPA, *M* ± *SD*	166.24 ± 64.72	143.62 ± 37.35	−1.24	0.30
Cognitive functioning, *M* ± *SD*	76.14 ± 10.05	74.38 ± 13.94	−0.37	0.71

Note: N/A = not applicable.

**Table 2:** Descriptive statistics of fundamental motor skills, MVPA, and cognitive functioning by groups at Time 1 and Time 2.

Variable	Control (*n* = 11)		Intervention (*n* = 20)		Total (*n* = 31)	
	*M*	*SD*	*M*	*SD*	*M*	*SD*
Locomotor skills (range 0–48)						
Time 1	29.73	7.96	25.40	8.02	26.94	8.14
Time 2	30.32	11.67	37.98	4.97	35.26	8.66
Gain	0.59	6.47	12.58	6.66	8.32	8.72
Object control skills (range 0–48)						
Time 1	27.05	10.79	24.68	9.73	25.52	10.01
Time 2	27.59	10.43	39.78	8.09	35.45	10.62
Gain	0.55	7.74	15.10	5.01	9.94	9.27
Total motor skills (range 0–96)						
Time 1	56.77	16.72	50.08	16.11	52.45	16.38
Time 2	57.91	21.48	77.75	12.29	70.71	18.51
Gain	1.14	12.13	27.68	9.35	18.26	16.46
MVPA (mins)						
Time 1	166.24	64.72	143.62	37.35	151.65	49.00
Time 2	155.17	47.43	170.06	42.66	164.78	44.22
Gain	−11.07	28.56	26.44	23.76	13.13	31.02
Cognitive functioning (range 0–100)						
Time 1	76.14	10.05	74.38	13.94	75.00	12.55
Time 2	78.03	13.96	73.54	17.38	75.13	16.15
Gain	1.89	13.35	−0.83	21.82	0.13	19.05

Note: *M* = mean, *SD* = standard deviation.

## Appendix A

**Table A1:** Twenty-four lessons for structured fundamental motor skill-focused afterschool program.

Session	Focused FMS	Game/Activity (Reference)	Students’ Learning Objectives
1	Running	**Traveling with running** (http://www.pecentral.org/lessonideas/ViewLesson.asp?ID=10872#.V_gt8_krLRY) **Running speed with dice** (http://www.pecentral.org/lessonideas/ViewLesson.asp?ID=10872#.V_gt8_krLRY) **Fisherman** (http://www.pecentral.org/lessonideas/ViewLesson.asp?ID=12882)	**P**: will be able to run with opposite arms and legs with elbows bent; **C**: will understand how to adjust running speed by following teachers’ signal and forms; **A**: will have fun and interact with diverse children in the class regardless of skill levels
2	Underhand rolling	**Rolling a ball to partner** (http://www.pecentral.org/lessonideas/ViewLesson.asp?ID=5#.V_gwBvkrLRY) **Strike**! (http://www.pecentral.org/lessonideas/ViewLesson.asp?ID=132742#.V_gv2vkrLRY) **Remove your wiffle balls** (http://www.pecentral.org/lessonideas/ViewLesson.asp?ID=2027#.V_gwRPkrLRY)	**P**: will be able to perform underhand roll by releasing the ball close to the floor; **C**: will understand how to underhand roll balls toward targets and partners by facing forward with opposite foot; **A**: will have fun and interact with diverse children in the class regardless of skill levels
3	Jumping	**Jump like Tigger the Tiger & Jump for distance** [Holt/Hale, S., & Hall, T. (2015). Lesson planning for elementary physical education (p. 77, 89)] **Hoop jumper** (http://www.pecentral.com/lessonideas/ViewLesson.asp?ID=341#.WDRpWy0rJhE)	**P**: will be able to jump in place and forward by flexing both knees, taking off and landing on both feet; **C**: will be able to recognize self-space and general space when jumping to different direction; **A**: will have fun and interact with diverse children in the class regardless of skill levels
4	Striking	**Striking with a paddle & Striking upward** [Holt/Hale, S., & Hall, T. (2015). Lesson planning for elementary physical education (p. 215)] **Striking off a tee** (http://www.pecentral.org/lessonideas/ViewLesson.asp?ID=370#.V_p4lfkrJhE)	**P**: will be able to strike a balloon and a ball with a plastic paddle; **C**: will understand how to swing from low to high and back to front properly with right amount of force; **A**: will follow instructions and not bump into others to ensure safety
5	Galloping	**Freeze** (http://www.brighthubeducation.com/elementaryschool-crafts-activities/123737-galloping-for-gross-motor-skill-development-activity-ideas/) **Cowboy riding a horse** (http://motherhood.modernmom.com/galloping-activities-children-15734.html) **Four colors and corners** (http://www.pecentral.org/lessonideas/ViewLesson.asp?ID=10872#.WC85UWorKUk)	**P**: will be able to lead with both dominant and non-dominant foot, and follow with the other foot; **C**: will remember to put their arms at waist level when driving forward; **A**: will have fun and interact with diverse children in the class regardless of skill levels
6	Catching	**Catch the UFO & Volcano** [Colvin, A.V., Markos, N., & Walker, P. (2016). Teaching fundamental motor skills (3rd ed) (p. 131 & 132). Human Kinetics] **Name balloon** (http://www.brighthubeducation.com/elementaryschool-crafts-activities/36533-outdoor-ball-games-for-elementaryschoolers/)	**P**: will be able to catch by putting hands in front of the body with flexed elbows; **C**: will understand to reach for the ball consistently when catching; **A**: will interact with other children with good attitudes when taking turns
7	Sliding	**Freeze** (http://www.brighthubeducation.com/elementaryschool-crafts-activities/123737-galloping-for-gross-motor-skill-development-activity-ideas/) **50 balls** (http://www.pecentral.com/lessonideas/ViewLesson.asp?ID=8298#.WC9Gj2orKUk) **Sliding scavenger hunt** (http://www.pecentral.org/lessonideas/ViewLesson.asp?ID=132926#.WC9I0orKUk)	**P**: will be able to step sideways with a slide to the trailing foot where both feet are off the ground; **C**: will recognize to turn their body sideways when sliding; **A**: will interact with other children with good attitudes when taking turns.
8	Kicking	**Inside of the food pass** (http://www.pecentral.org/lessonideas/ViewLesson.asp?ID=360#.WC8UYWorKUk) **Soccer bowling** (http://www.kidactivities.net/post/Games-for-Small-Groups-of-Kids.aspx) **Wall target** (http://www.livestrong.com/article/561042-kicking-games-for-kids/)	**P**: will be able to kick a ball with their instep; **C**: will recognize the concept of force to kick a ball harder or softer for varied distance; **A**: will interact with other children with good attitudes when taking turns
9	Hopping	**Hop in self-space & Hop in general space** [Holt/Hale, S., & Hall, T. (2015). Lesson planning for elementary physical education (p. 76)] **JUMP! Hopscotch** (http://www.pecentral.com/lessonideas/ViewLesson.asp?ID=9960#.WDR2Qi0rJhE)	**P**: will be able to hop in place and forward by taking off and landing on the same foot (both dominant and non-dominant foot); **C**: will be able to recognize self-space and general space when hoping to different directions; **A**: will follow instruction and music to engage in the activities with enjoyment
10	Dribbling	**Bounce and catch & Dribble with one hand** [Holt/Hale, S., & Hall, T. (2015). Lesson planning for elementary physical education (p. 189-190)] **Red light green light 3, 2, 1** (http://www.pecentral.com/lessonideas/ViewLesson.asp?ID=11122#.WDTpTS0rJhE)	**P**: will be able to dribble a ball with dominant hand and attempt a second contact; **C**: will understand how to remain in self-space when dribbling; **A**: will follow rules and not bump into others to ensure safety
11	Leaping	**Through the jungle** (http://www.pecentral.org/lessonideas/ViewLesson.asp?ID=11971#.V_guk_krLRY) **Adventure in the hula hoop cave** (http://www.pecentral.org/lessonideas/ViewLesson.asp?ID=340#.WC4r9rIrLRY) **Don’t fall off the cliff** (http://www.pecentral.org/lessonideas/ViewLesson.asp?ID=11971#.V_guk_krLRY)	**P**: will be able to take off on one foot and land on the opposite foot, with a longer period off the ground than running; **C**: will be able to recognize airborne time in leaping in order to perform in proper form; **A**: will have fun and interact with diverse children in the class regardless of skill levels
12	Overhand throwing	**Three points shoot** (http://www.pecentral.org/lessonideas/ViewLesson.asp?ID=8015#.V9ahUPkrKUk) **Hot potato** (http://www.pecentral.org/lessonideas/ViewLesson.asp?ID=1041#.WC88NrIrLRY) **Goal and score** (http://www.pecentral.org/lessonideas/ViewLesson.asp?ID=132844#.V_gvS_krLRY)	**P**: will be able to overhand throw by stepping opposite foot forward and following through; **C**: will understand how to overhand throw balls toward a target with an initial downward windup; **A**: will have fun and interact with diverse children in the class regardless of skill levels
13	Galloping & Sliding	**Slide, slide, and gallop** (https://www.wired.com/2009/08/simpleoutdoorplay/) **Sailors and sharks** (http://www.pecentral.org/lessonideas/ViewLesson.asp?ID=291#.WBahSy0rKUk) **Crab and horse** (http://www.pecentral.org/lessonideas/ViewLesson.asp?ID=10264#.WC9i42orKUk)	**P**: will be able to maintain a rhythmic pattern for four consecutive gallops/slides (to left and right); **C**: will be able to understand the different body orientations in galloping and sliding; **A**: will have good attitudes when interacting with other children and taking turns.
14	Catching & Kicking	**The launch board catch** (http://www.pecentral.org/lessonideas/ViewLesson.asp?ID=369#.V_qvS_krKUk) **Ghostbusters** (http://www.pecentral.org/lessonideas/ViewLesson.asp?ID=2175#.WDpmvLIrKUk)	**P**: will be able to catch from a 15-feet distance and kick a 20-feet distance ball; **C**: will understand how to use different body parts for catching and kicking; **A**: will interact with other children with good attitudes when taking turns
15	Jumping & Hopping	**Jump and hop over rivers** (http://www.pecentral.com/lessonideas/ViewLesson.asp?ID=2121#.V_pXGPkrKUk) **Shark attack jumping and landing** (http://www.pecentral.com/lessonideas/ViewLesson.asp?ID=340#.WDRqOy0rJhE)	**P**: will be able to alternate jump and hop forward by taking off and landing on the correct number of feet; **C**: will be able to recognize how much arm swing is needed to jump/hop over the ropes; **A**: will follow rules and not bump into others to ensure safety
16	Striking & Dribbling	**Cone Baseball** (http://www.pecentral.com/lessonideas/ViewLesson.asp?ID=15#.WDTvQS0rJhE) **Striking with bats & Dribbling choices** [Holt/Hale, S., & Hall, T. (2015). Lesson planning for elementary physical education (p. 225 & p. 190)]	**P**: will be able to strike and dribble a stationary ball with mature patterns; C: will understand how to remain in self-space when striking and dribbling; A: will follow rules and not bump into others to ensure safety
17	Running & Leaping	**Run, run, leap, leap** (http://www.pecentral.org/lessonideas/ViewLesson.asp?ID=11971#.WC448LIrLRY) **Treasure hunting** (https://www.wired.com/2009/08/simpleoutdoorplay/) **Catch me if you can 2** (http://www.pecentral.org/lessonideas/ViewLesson.asp?ID=9284#.WC45D7IrLRY)	**P**: will be able to forward reach with the arm opposite to the lead foot for running and leaping; **C**: will understand how to perform a leap off the ground longer than a run in general space; **A**: will have fun and interact with diverse children in the class regardless of skill levels
18	Underhand Rolling & Overhand throwing	**Roll and Throw** (https://www.wired.com/2009/08/simpleoutdoorplay/) **Goal and score** (http://www.pecentral.org/lessonideas/ViewLesson.asp?ID=132844#.V_gvS_krLRY) **Pass me the ball** (http://www.pecentral.org/lessonideas/ViewLesson.asp?ID=1041#.WCIYq2orLRY)	**P**: will be able to underhand roll and overhand throw at least a 20-feet distance with correct forms; **C**: will understand how to underhand roll and overhand throw balls toward targets using a straight pathway; **A**: will have fun and interact with diverse children in the class regardless of skill levels
19	Galloping & Sliding	**Snowflake game** (http://www.pecentral.org/lessonideas/Viewlesson.asp?ID=7322#.V_q6l_krKUk) **Sliding & galloping race** (http://www.pecentral.org/lessonideas/ViewLesson.asp?ID=5780#.WBq9uWorKUk) **Foam balls clean-up** (http://www.pecentral.org/lessonideas/ViewLesson.asp?ID=7063#.WC9paWorKUk)	**P**: will be able to maintain a rhythmic pattern for four consecutive gallops/slides (to left and right); **C**: will be able to understand the different speeds and body orientations for galloping and sliding; **A**: will have good attitudes when interacting with other children and taking turns.
20	Catching & Kicking	**Goalie kicking** (http://www.pecentral.org/lessonideas/ViewLesson.asp?ID=818#.WBq8sWorKUk) **Hungry hungry children** (http://www.pecentral.org/lessonideas/ViewLesson.asp?ID=12259#.WC9wumorKUk) **Ocean rescue** (http://www.pecentral.org/lessonideas/ViewLesson.asp?ID=10385#.WC9zgmorLRY)	**P**: will be able to catch and kick a ball with mature patterns; **C**: will understand how to remain in self-space when catching and kicking; **A**: will follow rules and not bump into others to ensure safety
21	Jumping & Hopping	**Corner-to-corner locomotors** [Holt/Hale, S., & Hall, T. (2015). Lesson planning for elementary physical education (p. 86)] **Jump/hop over Hot hoops** (http://www.pecentral.com/lessonideas/ViewLesson.asp?ID=3241#.V_pXHPkrKUk)	**P**: will be able to alternate jump and hop forward by taking off and landing on the correct number of feet; **C**: will be able to apply arm swing upward and outward when jumping/hopping over the hula hoops; **A**: will follow rules and not bump into others to ensure safety
22	Striking & Dribbling	**Dribble with head up** [Holt/Hale, S., & Hall, T. (2015). Lesson planning for elementary physical education (p. 191)] **Basketball tag & Three hit baseball** [ACHPER (2009). Fundamental motor skills module (p. 28 & p. 26)]	**P**: will be able to striking and dribble a stationary ball with mature patterns; **C**: will understand how to remain in self-space when striking and dribbling; **A**: will follow rules and not bump into others to ensure safety
23	Running & Leaping	**Follow me** (http://www.pecentral.org/lessonideas/ViewLesson.asp?ID=132921#.V_gs2_krLRY) **Escaping from the jungle** (http://www.pecentral.org/lessonideas/ViewLesson.asp?ID=11971#.V_guk_krLRY) **Frozen** (http://www.pecentral.org/lessonideas/ViewLesson.asp?ID=6321#.WCIaumorLRY)	**P**: will be able to forward reach with arms opposite to the lead foot for running and leaping; **C**: will understand how to perform a leap off the ground longer than a run; **A**: will have fun and interact with diverse children in the class regardless of skill levels
24	Underhand rolling & Overhand throwing	**Hit the walls** (http://www.pecentral.org/lessonideas/ViewLesson.asp?ID=347#.WCIXp2orLRY) **Hot potato 2** (http://www.pecentral.org/lessonideas/ViewLesson.asp?ID=1041#.WC88NrIrLRY) **Remove the bombs** (http://www.pecentral.org/lessonideas/ViewLesson.asp?ID=2027#.V_gwRPkrLRY)	**P**: will be able to perform underhand roll and overhand throw with opposite foot forward; **C**: will understand the differences between understand and overhand forms; **A**: will have fun and interact with diverse children in the class regardless of skill levels

Note: All lessons include 60 minutes of activities (i.e., warm-up, introductory and developmental activities, and cool-down); FMS = fundamental motor skills; P = psychomotor, C = cognitive, A = affective.
